# *In situ* immune response and mechanisms of cell damage in central nervous system of fatal cases microcephaly by Zika virus

**DOI:** 10.1038/s41598-017-17765-5

**Published:** 2018-01-08

**Authors:** Raimunda S. S. Azevedo, Jorge R. de Sousa, Marialva T. F. Araujo, Arnaldo J. Martins Filho, Bianca N. de Alcantara, Fernanda M. C. Araujo, Maria G. L. Queiroz, Ana C. R. Cruz, Beatriz H. Baldez Vasconcelos, Jannifer O. Chiang, Lívia C. Martins, Livia M. N. Casseb, Eliana V. da Silva, Valéria L. Carvalho, Barbara C. Baldez Vasconcelos, Sueli G. Rodrigues, Consuelo S. Oliveira, Juarez A. S. Quaresma, Pedro F. C. Vasconcelos

**Affiliations:** 1 000 0004 0620 4442 Departamento de Arbovirologia e Febres Hemorrágicas, Instituto Evandro Chagas, Ananindeua, Pará Brazil; 2 000 0004 0620 4442 Programa de Pós-Graduação em Virologia, Instituto Evandro Chagas, Ananindeua, Pará Brazil; 3 000 0004 0620 4442 Departamento de Patologia, Instituto Evandro Chagas, Ananindeua, Pará Brazil; 4 Laboratório Central de Saúde Pública, SES do Ceará, Fortaleza, Ceará Brazil; 5 Laboratório Central de Saúde Pública, SES do Rio Grande do Norte, Natal, Rio Grande do Norte Brazil; 6 Universidade do Estado do Pará, Belém, Pará Brazil; 7 Núcleo de Medicina Tropical, Universidade Federal do Pará, Belém, Pará Brazil

## Abstract

Zika virus (ZIKV) has recently caused a pandemic disease, and many cases of ZIKV infection in pregnant women resulted in abortion, stillbirth, deaths and congenital defects including microcephaly, which now has been proposed as ZIKV congenital syndrome. This study aimed to investigate the *in situ* immune response profile and mechanisms of neuronal cell damage in fatal Zika microcephaly cases. Brain tissue samples were collected from 15 cases, including 10 microcephalic ZIKV-positive neonates with fatal outcome and five neonatal control flavivirus-negative neonates that died due to other causes, but with preserved central nervous system (CNS) architecture. In microcephaly cases, the histopathological features of the tissue samples were characterized in three CNS areas (meninges, perivascular space, and parenchyma). The changes found were mainly calcification, necrosis, neuronophagy, gliosis, microglial nodules, and inflammatory infiltration of mononuclear cells. The *in situ* immune response against ZIKV in the CNS of newborns is complex. Despite the predominant expression of Th2 cytokines, other cytokines such as Th1, Th17, Treg, Th9, and Th22 are involved to a lesser extent, but are still likely to participate in the immunopathogenic mechanisms of neural disease in fatal cases of microcephaly caused by ZIKV.

## Introduction

Zika virus (ZIKV; genus *Flavivirus*, family *Flaviviridae*) was isolated in 1947 from *Macaca mulatta* in the Zika Forest of Uganda^1^ and, similar to other flaviviruses, can cause lesions in the lung, kidney, heart, liver, and brain^2^.

In view of the severity of presentation, many studies are currently trying to characterize the relationship of ZIKV infection with the development of Guillain-Barré syndrome and microcephaly, as well as the mechanisms underlying sexual and vertical transmission of the virus^1,3,4^. However, at the CNS level, there are still many gaps in the current understanding of the mechanisms that induce ZIKV infection-associated microcephaly. Some studies have already demonstrated that underlying neuronal cell death is directly related to microcephaly, especially because of the production of large amounts of caspase 3 in cortical neurons^5,6^. However, with respect to the immune response that occurs in the brain of newborns with microcephaly, no studies have yet used this approach. In particular, studies investigating the *in situ* immune response in the tissue microenvironment are lacking. This tissue immune response is important for understanding of the immunopathogenesis of lesions, as this local response is intimately related to the mechanisms of immune-mediated tissue damage.

Because the CNS is an immunologically privileged site and in view of the intense selectivity of the blood-brain barrier in terms of kinetics of cell and cytokine flow between blood and tissue, investigation of the *in situ* immune response profile and neuronal cell damage mechanism is important for understanding the pathogenesis of microcephaly by ZIKV. Therefore, by recognizing that this relationship can provide new insights into the immunopathogenesis of the disease, the present study investigated the *in situ* immune response profile and mechanism of neuronal cell damage in stillborn infants and newborns infected with ZIKV who died.

## Results

### Patients

This study was performed on brain tissue samples from 10 fatal microcephaly cases. The population consisted of eight newborns (two female and six male), with a lifetime ranging from 2 hours to 27 days (median one day) and two stillbirths (female and male). Five patients (four female and one male) were used as control samples in the investigation. Additional information on the cases can be found in Supplementary Table S7.

### Description of the main histopathological alterations found in newborns with microcephaly caused by Zika virus

**Figure 1:**
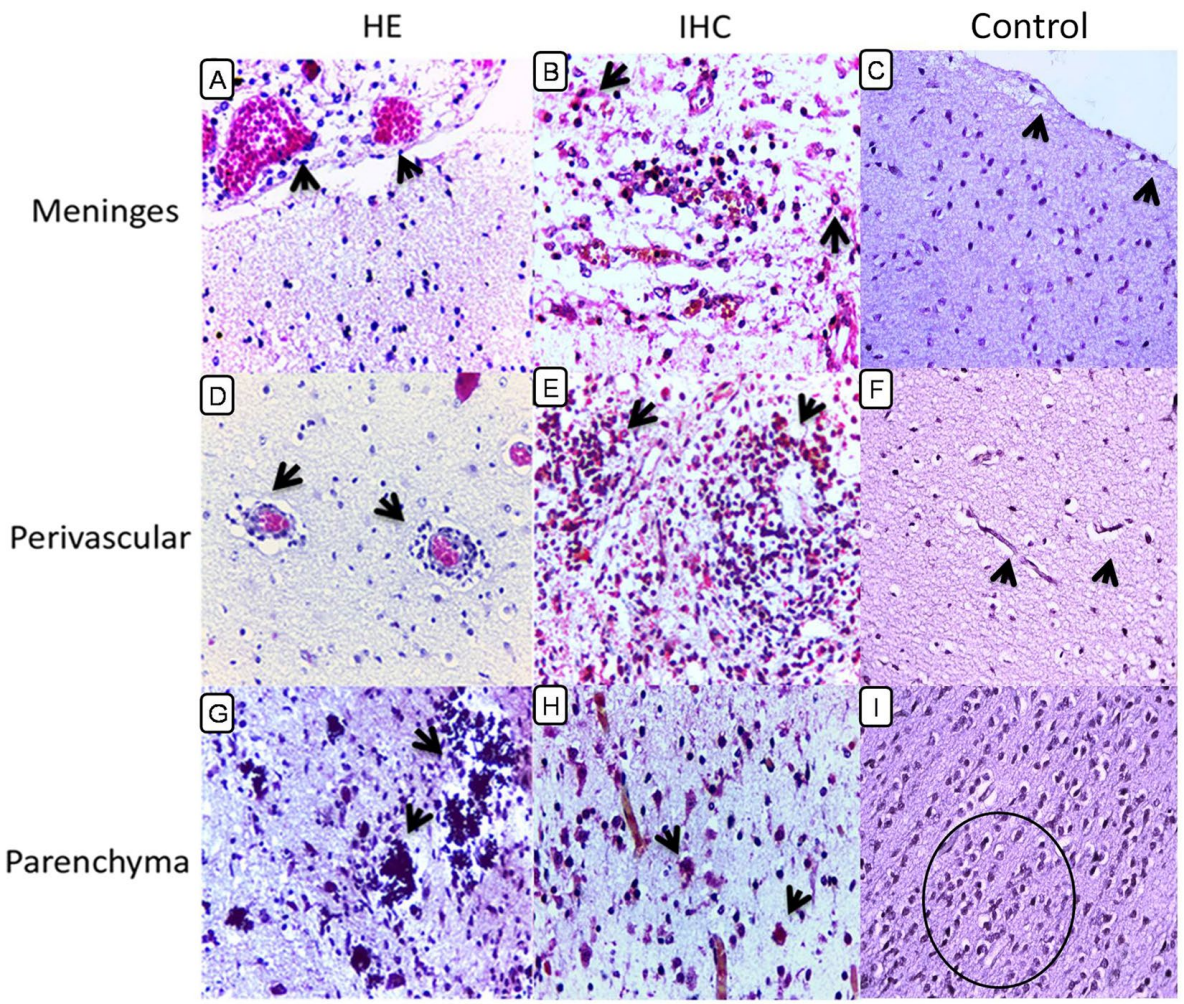
Histopathological and immunohistochemical (IHC) aspects of fatal ZIKV-associated microcephaly cases and controls. (**A**) (Case 6): Viral meningoencephalitis with vasocongestion and surrounding inflammatory infiltrate (arrow). (**B**) (Case 6): Immunostaining of ZIKV antigens in cells of the inflammatory infiltrate in the meninges (arrow). (**C**) (Case 15): Preserved meninges and IHC-negative to control (arrow). (**D**) (Case 6): Infiltration of mononuclear cells in the perivascular space (arrow). (**E**) (Case 8): Gliosis and immunostaining inflammatory cell infiltrate (arrow). (**F**) (Case 15): Preserved capillaries without inflammatory infiltrate and IHC-negative to control (arrow). (**G**) (Case 8): Calcification (arrow) in the neural parenchyma. (**H**) (Case 5): Neuronal necrosis (arrow) with ZIKV antigens in the neural parenchyma. (**I**) (Case 15): Parenchymal neural preservation and IHC-negative to control (circle) (magnification: 400×).

The histopathological features of the tissues samples were characterized by meningeal, perivascular, and parenchymatous changes. Vascular congestion and inflammatory infiltrates consisting mainly of lymphocytes and macrophages were observed in the meninges, suggestive of viral meningitis (Fig. 1A,B). The perivascular environment exhibited congestion of variable intensity and inflammatory infiltration of mononuclear cells (Fig. 1D) filling the Virchow-Robin space. Analysis of the intraparenchymatous environment showed alterations of varying intensity including gliosis (Fig. 1E), calcifications (Fig. 1G), neuronophagy, neuronal necrosis (Fig. 1H), microglial nodules, vacuolization, and neuronal disappearance.

### Immunohistochemical evaluation

The observation of a brown color in the tissue fragments was defined as positive immunostaining for the different processes evaluated, including the mechanisms of tissue damage and the host immune response in tissue damaged by ZIKV infection.

### Apoptosis

**Figure 2:**
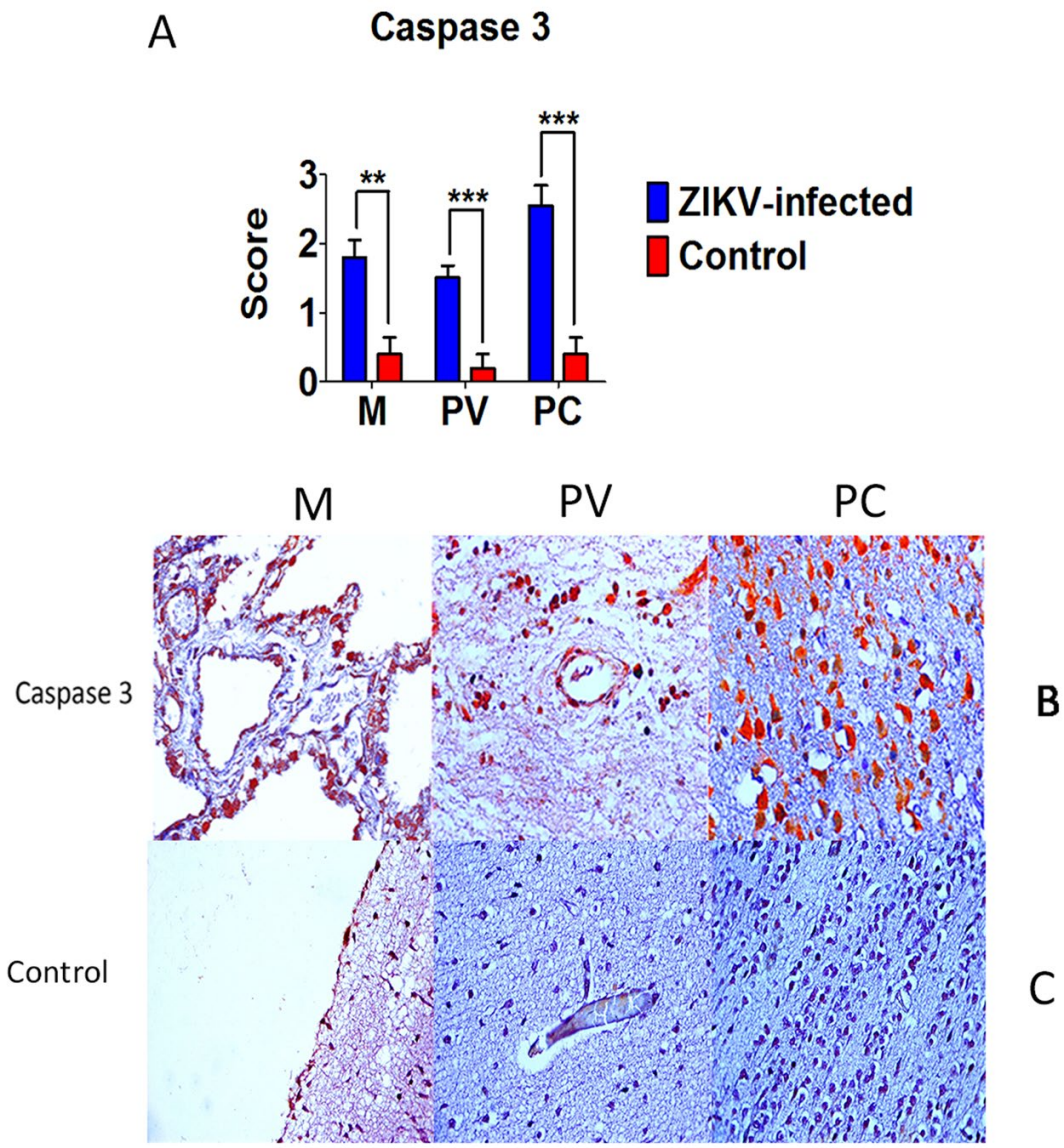
Semiquantitative analysis and representative **i**mmunohistochemical pattern in meninges (M), perivascular region (PV) and parenchyma (PC) in fatal Zika microcephaly cases and control. (**A**) Expression of caspase 3. (**B**) Immunolabeling for caspase 3 (B-M and B-PV: Case 7; B-PC: Case 2). (**C**) Negative control (C-M, C-PV, and C-PC: Case 15) (magnification: 400×) (**p < 0.005, ***p < 0.0005).

Caspase 3 immunostaining in the three compartments analyzed (meninges, perivascular space, and parenchyma) differed significantly compared to control. The increase in caspase 3 expression was particularly marked in the parenchyma, especially in cases of neuronal damage in the cortex in which intense staining was observed in vacuolated and apoptotic neurons (Supplementary Table S3) (Fig. 2).

### Characterization of the cell infiltrate

**Figure 3:**
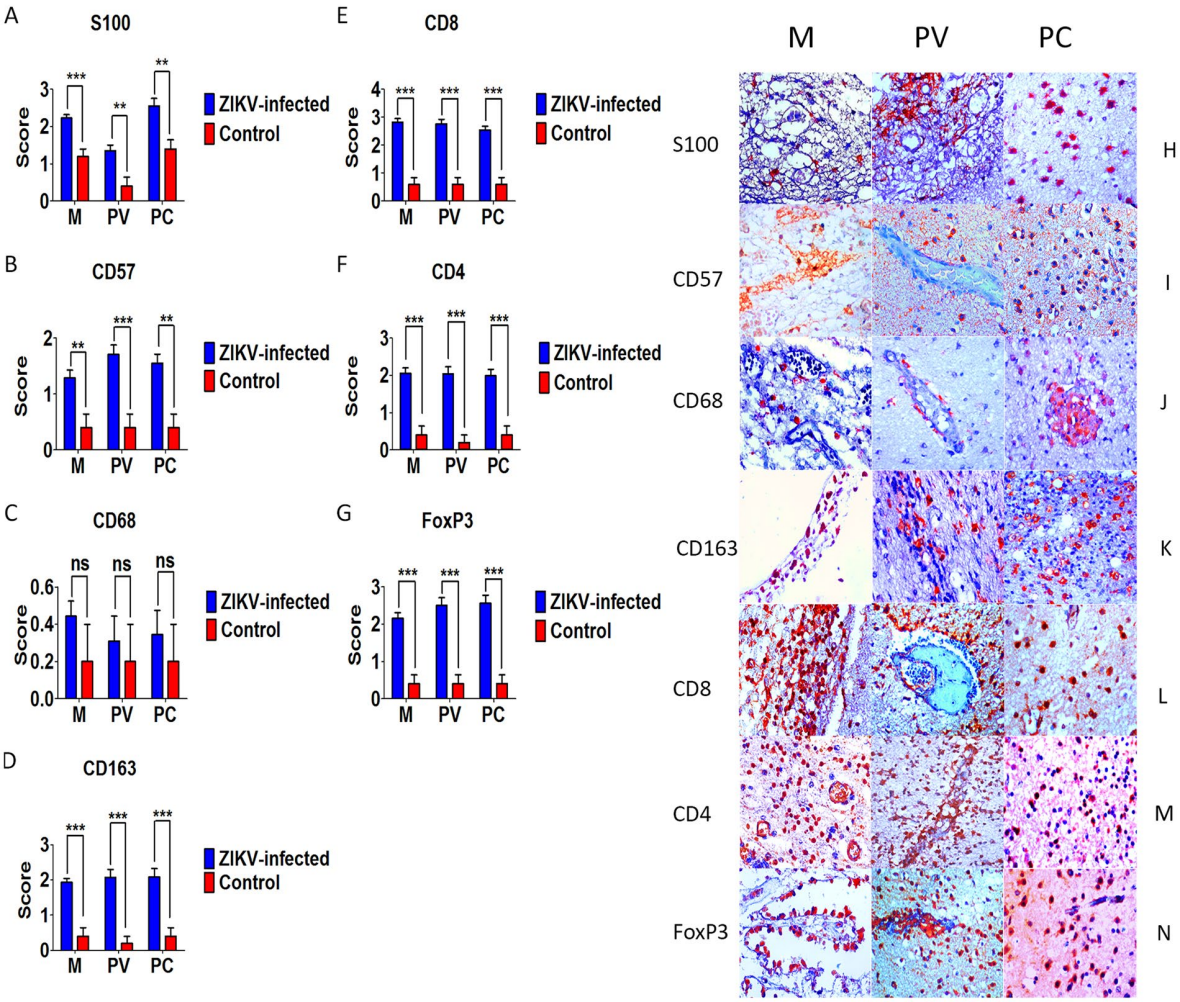
Semiquantitative analysis and Immunohistochemical pattern in meninges (M), perivascular region (PV), and parenchyma (PC) in fatal Zika microcephaly cases. (**A**) Expression of S100. H Immunolabeling for S100 (H-M and H-PV: Case 6; H-PC: Case 5). (**B**) Expression of CD57. I Immunolabeling for CD57 (I-M: Case 6; I-PV and I-PC: Case 7). (**C**) Expression of CD68. J Immunolabeling for CD68 (J-M: Case 6; J-PV and J-PC: Case 5). (**D**) Expression of CD163. K Immunolabeling for CD163 (K-M: Case 8; K-PV and K-PC: Case 5). (**E**) Expression of CD8. L Immunolabeling for CD8 (L-M: Case 6; L-PV and L-PC: Case 7). (**F**) Expression of CD4. M Immunolabeling for CD4 (M-M: Case 6; M-PV and M-PC: Case 7). (**G**) Expression of Foxp3. N Immunolabeling for Foxp3 (N-M: Case 8; N-PV and N-PC: Case 7) (magnification: 400×) (ns: not statistically significant, *p < 0.05, **p < 0.005, ***p < 0.0005).

Characterization of the phenotype of the local inflammatory infiltrate revealed the presence of antigen-presenting cells and astrocytes (S100), natural killer (NK) cells (CD57), M1 macrophages/microglia (CD68), M2 macrophages/microglia (CD163), T lymphocytes (CD8+, CD4+), and regulatory T (Treg) cells (FoxP3) (Supplementary Table S3) (Fig. 3).

S100-positive cells were observed in the three compartments analyzed and a significant difference was found compared to the control (Fig. 3A). In cases in which thickened meninges and marked vascularization were observed, S100 immunostaining was associated with the presence of antigen-presenting cells. S100-positive cells were found in the parenchyma and perivascular space, as well as in areas of the cortex characterized by neuronal depopulation. In the neural parenchyma, intense S100 immunostaining was observed in gemistocytic astrocytes (Fig. 3H).

In the three compartments, the number of CD57 + NK cells was increased in the mononuclear inflammatory infiltrate (Fig. 3B,I). Differences were observed in the mean number of CD68 and CD163-positive cells, which characterize the presence of M1 and M2 macrophages/microglia, respectively. There was no significant difference in the expression of CD68 compared to the control (Fig. 3C). In contrast, CD163 expression was increased in cases with symptoms of meningoencephalitis, accompanied by intense vascular proliferation and focal necrosis in the cortical layer (Fig. 3D,K).

Semiquantitative analysis of T lymphocyte subpopulations (CD8, CD4) and Treg cells (FoxP3) showed increased expression of these markers in the three compartments, especially in areas exhibiting a dense mononuclear infiltrate (Fig. 3E,F,G,L,M and N).

### Cytokine profile and factors involved in the inflammatory response

**Figure 4:**
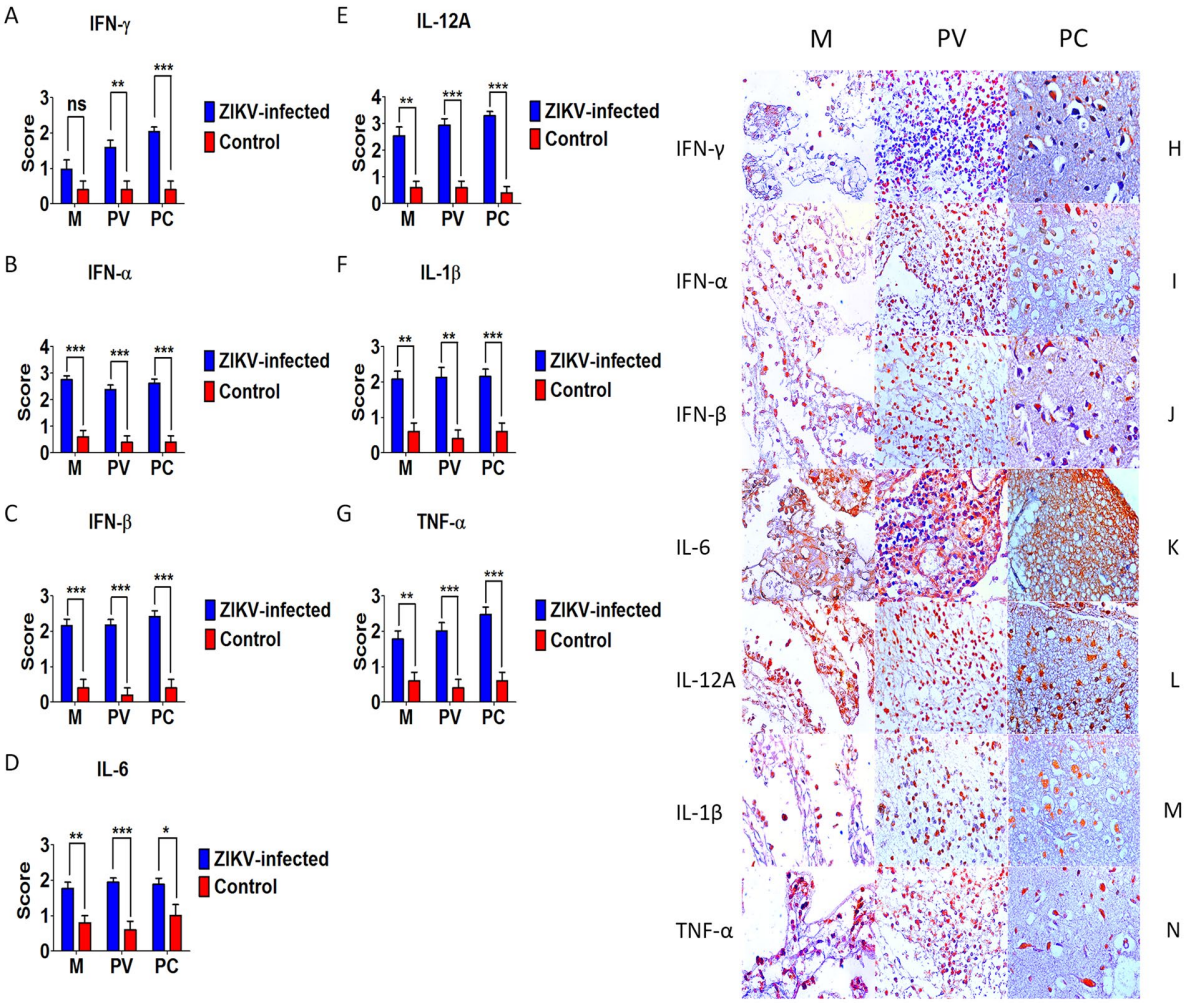
Semiquantitative analysis and Immunohistochemical pattern in meninges (M), perivascular region (PV), and parenchyma (PC) in fatal Zika microcephaly cases. (**A**) Expression of IFN-γ. H Immunolabeling for IFN-γ (H-M and H-PV: Case 8; H-PC: Case 9). (**B**) Expression of IFN-α. I Immunolabeling for IFN-α (I-M: Case 8; I-PV: Case 9; I-PC: Case 7). (**C**) Expression of IFN-β. J Immunolabeling for IFN-β (J-M and J-PV: Case 8; J-PC: Case 9). (**D**) Expression of IL-6. K Immunolabeling for IL-6 (K-M: Case 9; K-PV: Case 8; K-PC: Case 7) (**E**) Expression of IL-12A. L Immunolabeling for IL-12A (L-M and L-PV: Case 8; L-PC: Case 7). (**F**) Expression of IL-1β. M Immunolabeling for IL-1β (M-M: Case 8; M-PV and M-PC: Case 7). (**G**) Expression of TNF-α. N Immunolabeling for TNF-α (N-M and N-PV: Case 8 N-PC: Case 9) (magnification: 400×) (ns: not statistically significant, *p < 0.05, **p < 0.005, ***p < 0.0005).

**Figure 5:**
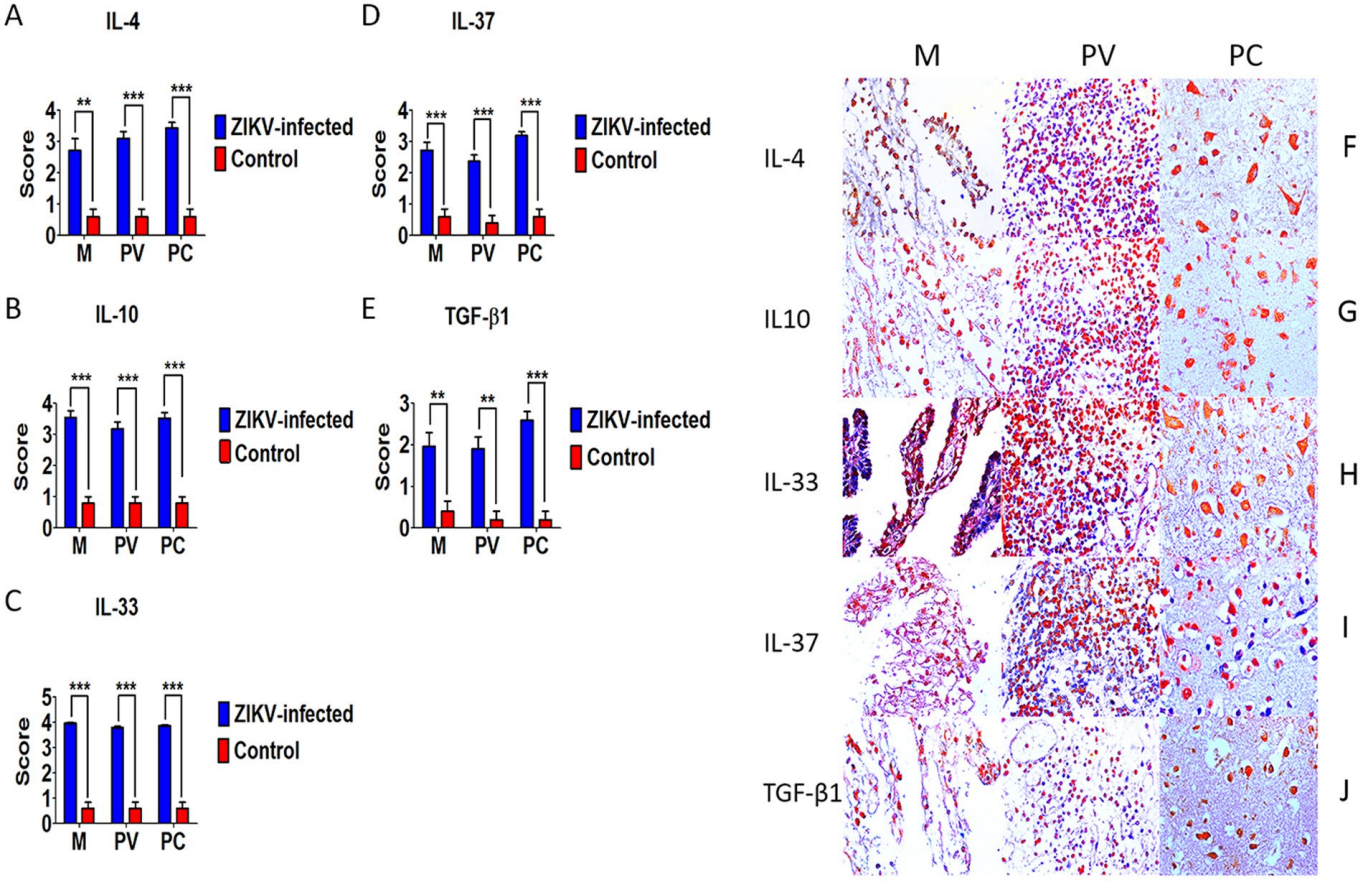
Semiquantitative analysis and immunohistochemical staining in meninges (M), perivascular region (PV), and parenchyma (PC) in fatal Zika microcephaly cases. (**A**) Expression of IL-4. F Immunolabeling for 4 (F-M and F-PV: Case 8; F-PC: Case 9). (**B**) Expression of IL-10. G Immunolabeling for 10 (G-M and G-PV: Case 8; G-PC: Case 4). (**C**) Expression of IL-33. H Immunolabeling for IL-33 (H-M: Case 7; H-PV: Case 8; H-PC: Case 9). (**D**) Expression of IL-37. I Immunolabeling for IL-37 (I-M and I-PV: Case 8; I-PC: Case 7). (**E**) Expression TGF-β1. J Immunolabeling for TGF-β1 (J-M: Case 8; J-PV: Case 7; J-PC: Case 9) (magnification: 400×) (**p < 0.05, ***p < 0.0005).

**Figure 6:**
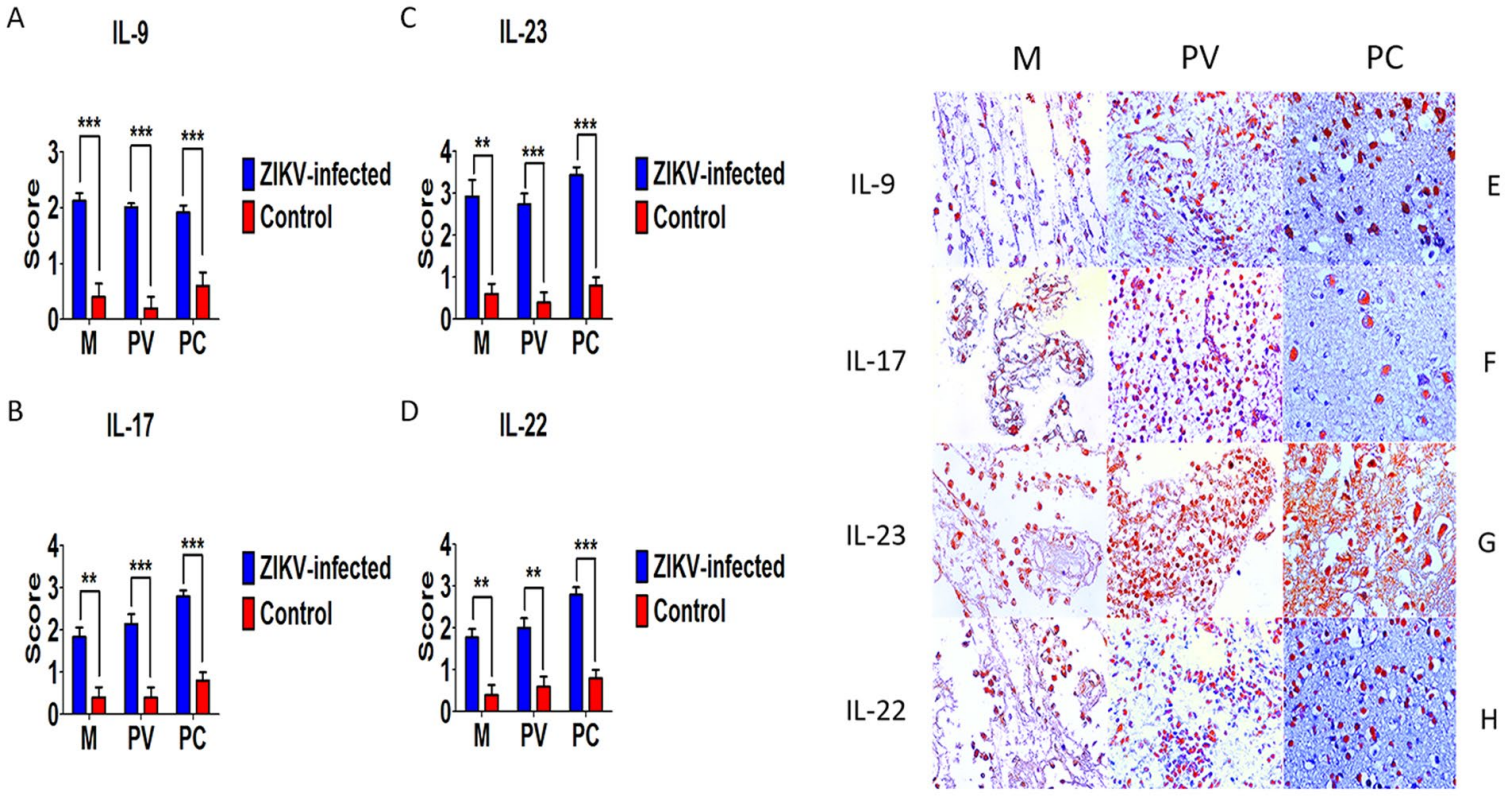
Semiquantitative analysis and immunohistochemical staining in meninges (M), perivascular region (PV), and parenchyma (PC) in fatal Zika microcephaly cases. (**A**) Expression of IL-9. F Immunolabeling for IL-9 (F-M and F-PV: Case 8 F-PC: Case 4). (**B**) Expression of IL-17. G Immunolabeling for IL-17 (G-M: Case 8; G-PV and G-PC: Case 7). (**C**) Expression of IL-23. H Immunolabeling for IL-23 (H-M: Case 8 and H-PV: H-PC: Case 9), (**D**) Expression of IL-22. H Immunolabeling for IL-22 (H-M: Case 8; H-PV: Case 9; H-PC: Case 2) (magnification: 400×) (**p < 0.005, ***p < 0.0005).

**Figure 7:**
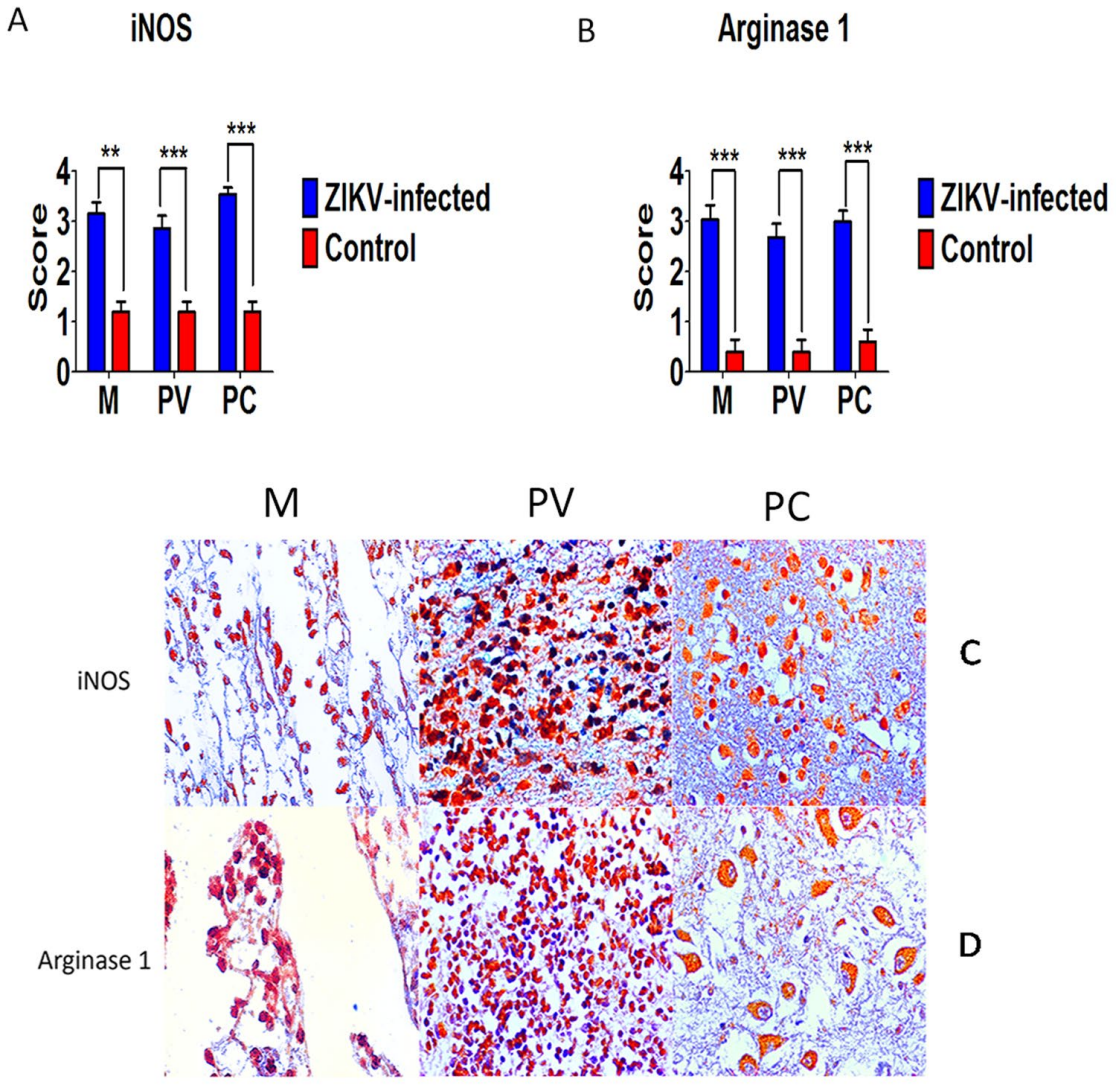
Semiquantitative analysis and immunohistochemical staining in meninges (M), perivascular region (PV), and parenchyma (PC) in fatal Zika microcephaly cases. (**A**) Expression of iNOS. (**C**) Immunolabeling for iNOS (C-M: Case 8; C-PV: Case 7; C-PC: Case 1). (**B**) Expression of arginase 1. (**D**) Immunolabeling for arginase 1 (D-M and D-PV: Case 8; D-PC: Case 9) (magnification: 400×) (**p < 0.05, ***p < 0.0005).

Analysis of the profile of cytokines and inflammatory factors in the samples showed expression of IFN-γ, IFN-α, IFN-β, IL-6, IL-12A, IL-1β, and TNF-α (Supplementary Table S3) (Fig. 4); IL-4, IL-10, IL-33, IL-37, and TGF-β1 (Supplementary Table S3) (Fig. 5); IL-9, IL-17, IL-23, and IL-22 (Supplementary Table S3) (Fig. 6); and iNOS and arginase 1 (Supplementary Table S3) (Fig. 7). There was a predominance of Th2 markers accompanied by proinflammatory cytokines (Figs 4 and 5), iNOS, and arginase 1, which exhibited increased expression in the three compartments when compared to control (Fig. 7). In the meninges, cellular expression was prominent in the mononuclear inflammatory infiltrate, and the perivascular space showed a similar immunostaining pattern. Immunoexpression of iNOS, arginase 1, and cytokines, especially IL-4, IL-10, IL-12A, IL-23, and IL-33, was more intense in the brain parenchyma. Expression was observed in the mononuclear inflammatory infiltrate, in areas containing vacuolated neurons, in areas of necrosis, and in apoptotic cells.

## Discussion

Zika virus is an infectious agent that has raised global concern because infection can cause fetal death and newborn sequelae including microcephaly^1^. The present results in stillbirth and neonate deaths caused by ZIKV showed an intrinsic and complex relationship between the immune response and the occurrence of cell damage. In this study, the main types of cell death were necrosis and apoptosis. We observed that the immunoexpression of IL-4, IL-10, IL-33, iNOS, and arginase 1 was increased in cases in which neuronal necrosis in the cortical layer was very intense. Among these markers, IL-33 may be a key factor to further elucidate the genesis of neuronal necrosis, as this cytokine participates directly in the process of necroptosis^7^.

Apoptosis is probably the key type of cell death involved in the development of ZIKV-induced CNS injury. In the present study, caspase 3 expression was increased, especially in the parenchyma, comprising areas of the cerebral cortex (Fig. 2A,B). These findings corroborate the results of *in vitro* experimental models that suggest apoptosis to be a major factor in neuronal cell death and reduced brain mass in humans^8,9^. In addition, the increased caspase 3 expression, especially in the parenchyma, was positively correlated with the expression of a set of cytokines that are directly or indirectly associated with the phenomenon of apoptotic cell death (Supplementary Table S6).

The phenotype of the cell-mediated immune response to ZIKV in the three compartments consisted of antigen-presenting cells and astrocytes (S100), NK cells (CD57), M1 macrophages/microglia (CD68), M2 macrophages/microglia (CD163), T lymphocytes (CD4+, CD8+), and Treg cells (FoxP3). Immunostaining for S100 in the meninges and perivascular space indicated that perivascular astrocytes and antigen-presenting cells may participate actively in the neuroinflammatory response. Several studies on flaviviruses have reported a direct association of antigen-presenting cells with the induction of neurodegenerative processes due to neuroinflammation^10,11^. In an experimental model, ZIKV was found to infect large numbers of astrocytes present in the cortical and subcortical layers^12,13^. An interesting finding is that the infection of astrocytes was intimately related to increased expression of AXL^14,15^. Another aspect that has been discussed regarding the prolonged infection of astrocytes is that the persistence of ZIKV can lead to an increase in viral load in the cortical layer and to infection of additional cortical cells^16^. Furthermore, studies have demonstrated that loss of astrocytes in the cortex can induce the formation of an inflammatory process, generalized cell death, and a reduction in the density of glial and neuronal cell populations. This eventually results in the development of calcifications, which are common findings in cases of congenital infection and microcephaly due to ZIKV^16,17^.

Another aspect to be considered in the pathogenesis of neural infection with ZIKV is the role of M1 and M2 macrophages/microglia. In the three compartments of ZIKV-affected brains, we observed elevated expression of CD163 compared to the control (Fig. 3D). Recent studies have shown that M2 microglia and M2 macrophages express groups of markers that participate in the immunosuppressive response, including IL-4, IL-10, TGF-β1, IL-33, IL-37 and arginase 1^14,15,18^. In cases of microcephaly, the expression of CD163 is important because in flaviviruses, the scavenger receptor CD163 is one of the receptors used by these viruses to enter the cell^19^.

In the present study, the expression of CD57, an NK cell marker, was increased in the three compartments (Fig. 3B). In flaviviruses, other studies have demonstrated that NK cells participate in the antiviral response, inducing the production of IFN-γ, a cytokine that inhibits viral replication^20,21^. Correlation analysis revealed different behavior of NK cells in the three compartments. In the meninges, IL-33, IL-37, and TGF-β1 appeared to negatively regulate the response of NK cells (Supplementary Table S4). In contrast, in the infiltrate at the perivascular space and parenchyma, NK cells were possibly actively involved in the induction of apoptosis (via caspase 3) and production of TNF-α, IFN-α, and IFN-β (Supplementary Tables S5 and S6).

With respect to adaptive immunity, the increased expression of CD8+, CD4+ and FoxP3 in the three compartments suggests that the response of cytotoxic T lymphocytes and helper T lymphocyte subpopulations play a key role in the specific immune response to ZIKV. Indeed, among flaviviruses previous studies have shown that CD8 T lymphocytes can induce protective immunity against West Nile virus, dengue virus and yellow fever virus^22,23^. In studies on primates, infiltration of CD8 T lymphocytes into the parenchyma and perivascular space contributed to the development of neurotoxicity^24^. Other approaches have shown that CD8 T lymphocytes participate directly in the process of apoptosis, i.e., in the production of TNF-α, TRAIL, and caspase 3^25,26^.

Regarding T helper lymphocyte subpopulations, it was observed that the CD4 T lymphocyte response was associated with a large panel of cytokines, but there was a clear predominance of a Th2 (Fig. 5) profile represented by high immunoexpression of IL-33 when compared to the other cytokines. However, increased expression of Th1, Treg, Th9, Th17, and Th22 cytokines was also observed when compared to controls (Figs 4–6). In general, the response of Th1 lymphocytes was confirmed by immunostaining for IFN-γ, IFN-α, IFN-β, IL-6, IL-12A, IL-1β, and TNF-α (Fig. 4). The increase of proinflammatory cytokines in the medium, suggests a response of proteins that might be associated with the induction of mechanisms of cellular damage, as these are the main mediators that trigger neurotoxicity and the production of reactive oxygen and nitrogen intermediates, which are directly involved in the cell death. The response of IFN-γ, IFN-α, and IFN-β is probably related to the development of a classical antiviral response against ZIKV. Indeed, recent studies have shown that expression of IFN-α and IFN-β is crucial to inhibit viral replication^27,28^. Together with IL-6, these cytokines may induce a pattern of acute inflammation that causes neurotoxicity probably associated with infection^29^. The increased expression of interferons is supported by the presence of IL-12A, a cytokine that classically induces the production of IFN-γ, which, in turn, may modulate the activity of microglia in response to the presence of ZIKV^30^. Finally, the expression of IL-1β and TNF-α could be also involved in the mechanism of cell death by apoptosis, as these cytokines are related to the activation of death receptors and the formation of inflammasomes, respectively^27,29^.

Immunostaining for IL-4, IL-10, IL-33, IL-37, and TGF-β1 characterized the presence of a Th2 response, which predominated in the cases studied (Fig. 5). The occurrence of a predominantly immunosuppressive local response has been reported for other presentations of viral encephalitis and might be related to the simultaneous development of a suppressive and antiviral response, which is necessary for elimination of the infectious agent without causing major inflammatory damage^30–33^.

Cytokines such as IL-33 exert multiple actions and are related to pyroptosis, activation of inflammasomes, and endoplasmic reticulum stress, which can all lead to cellular damage^34,35^. The involvement of IL-33 in cell death due to necrosis and apoptosis and in the induction of a M2 microglia response indicates that it is intimately related to the pathogenesis of ZIKV, inducing cell death and creating a more favorable environment for viral replication by activating M2 macrophages.

This anti-inflammatory environment, which is probably intended to protect the parenchyma from the deleterious effects of a potentially neurotoxic immune response, may be supported by the activity of Treg lymphocytes. Some studies have suggested a role of Treg lymphocytes in flavivirus infections, in which these cells contribute to the pathogenesis of acute infections caused by these viruses^36,37^.

Immunoexpression of Th9, Th17, and Th22 cytokines was observed in the samples examined (Fig. 6). These cytokines seem to be involved in specific pathogenic events during evolution of neonatal ZIKV disease. In this respect, IL-9 can increases the production of histamine and glutamate in cortical neurons, contributing to the induction of excytotoxicity and consequently to neuronal apoptosis^38^. Th17 cytokines such as IL-17 and IL-23 can induce the production of reactive oxygen and nitrogen intermediates, and equally lead to cell damage and cell death^39^. IL-22 is related to the recruitment of inflammatory cells. In addition, IL-22 modulates the production of CXCR2 against flaviviruses with tropism for CNS cells. Specifically, in the case of West Nile virus, IL-22 is a key component in the formation and development of encephalitis by potentiating the neuroinflammatory process^40,41^.

Analysis of enzymes that characterize the possible role of M1 and M2 macrophages and microglia in the CNS demonstrates a strong cellular stress response. iNOS and arginase 1 are antagonistic enzymes that compete for L-arginine, which serves as a substrate for the synthesis of L-citrulline, leading to the production of free radicals and of proline, and inducing cell division, collagen synthesis, and production of growth factors^42^. During the neuroinflammatory response, the expression of iNOS might be directly associated with the presence of IL-1β, IL-6, IL-12A, TNF-α, IFN-γ, IFN-α, IFN-β, IL-17, and IL-23. In contrast, because of immunosuppressive mechanisms, the production of arginase 1 could be directly related to the response of IL-4, IL-10, IL-33, IL-37, and TGF-β1.

**Figure 8:**
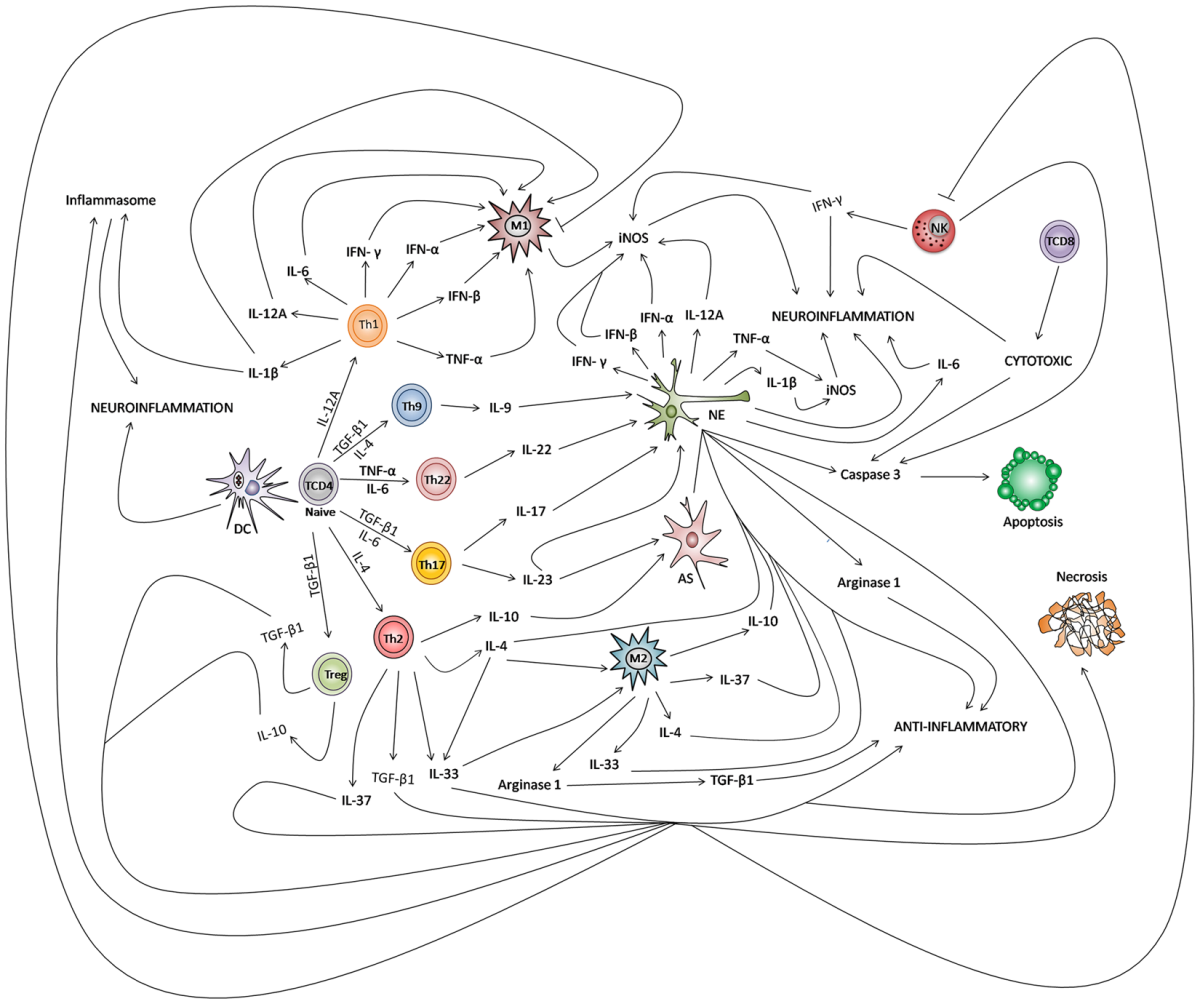
Integrated view of the *in situ* immune response in fatal cases of microcephaly caused by ZIKV. In view of the complexity of the immune response in the central nervous system, initially dendritic cells (DC) may trigger the development of neuroinflammation or present the viral antigen to naive TCD4 lymphocytes that can differentiate into several subpopulations of T helper lymphocytes (Th1, Th2, Th9, Th17, Th22, and Treg) that produce a series of cytokines modulating the activity of M1 and M2 microglia, astrocytes (AS), and neurons (NE). The production of pro-inflammatory (IL-1β, IL-6, IL-9, IL-17, IL-22, IL23, TNF-α, IFN-γ, IFN-α, and IFN-β) and anti-inflammatory cytokines (IL-4, IL-10, IL-33 IL-37, and TGF-β1) by these cells induces the neuroinflammatory or immunosuppressive response by increasing the production of iNOS or arginase 1. In the context of cellular stress, cytokines such as IL-4, IL-10, IL-33, IL-37, and TNF-α as well as other immune factors contribute directly to necrosis and apoptosis, regulating the production of caspase 3. With regard to the inflammasome, probably IL-1β and IL-33 induce neuroinflammation. NK cells participate in the apoptotic response and neuroinflammation, inducing IFN-γ production. In negative regulation, the immunosuppressive effect of anti-inflammatory cytokines inhibits the activity of M1 microglia as well as NK cells. In the cytotoxic response produced by TCD8 lymphocytes, these cells participate in mechanisms that lead to neuroinflammation as well as apoptosis.

The present results thus demonstrate that the *in situ* immune response against ZIKV in the CNS of newborns is highly complex. Despite the predominant expression of Th2 cytokines, other cytokines such as Th1, Th17, Treg, Th9, and Th22 cytokines are involved to a lesser extent, but are still likely to participate in the immunopathogenic mechanisms of neural disease. The cell response comprises CD4+ and CD8+ T cells, Treg cells, NK cells, M1/M2 macrophages, and antigen-presenting cells. The involvement of the three CNS compartments studied leads to ZIKV meningitis and encephalitis. The mechanism of neuronal cell death includes necrosis and apoptosis induced by different immune factors and probably by the action of the virus itself. Microglial cells play an important role by creating an inflammatory environment rich in reactive oxygen and nitrogen intermediates, inducing cell damage and cell death. Further studies, including in experimental models, are needed to elucidate in detail the pathogenetic mechanisms and the immune factors involved in the host response against CNS infection by ZIKV. Finally, Fig. 8 summarizes the most important interactions in the immune response of the CNS during fatal ZIKV microcephaly in light of the existing immunologic tools.

## Materials and Methods

### Ethics Statement

Biological samples of patients were obtained and processed in the context of the emergency definition of the Ministry of Health during surveillance activities of the ZIKV epidemic in Brazil. This study was approved (opinion number 1.888.946) by the Research Ethics Committee (CEP) of the Evandro Chagas Institute (IEC). All steps and methods of the study followed the recommendations established by legislation in force in Brazil.

### Patients, samples, and diagnostic confirmation

Brain tissue samples were collected from 15 cases. Among them, 10 fatal cases with microcephaly (eight newborns and two stillbirths) had positive results for ZIKV by RT-qPCR and/or immunohistochemistry^2,43^ and five patients (two newborns and three stillbirths) whose laboratory investigation for arboviruses (ZIKV, DENV, and CHIKV) was negative had preserved neural architecture.

For histopathological analysis, 5 µm sections were cut from paraffin-embedded tissue samples, stained with hematoxylin-eosin^44^, and subjected to immunohistochemistry using a panel of antibodies. The panel of antibodies tested is shown in Supplementary Table S1.

### Immunohistochemistry

The streptavidin alkaline phosphatase method was adapted to detect the viral antigen using a polyclonal anti-ZIKV antibody produced at the Evandro Chagas Institute^2^. The biotin-streptavidin peroxidase method was used for immunostaining of tissues with antibodies specific for each marker studied. First, the tissue samples were deparaffinized in xylene and hydrated in a decreasing ethanol series (90%, 80%, and 70%). Endogenous peroxidase was blocked by incubating the sections in 3% hydrogen peroxide for 45 min. Antigen retrieval was performed by incubation in citrate buffer, pH 6.0, or EDTA, pH 9.0, for 20 min at 90 °C. Nonspecific proteins were blocked by incubating the sections in 10% skim milk for 30 min. The histological sections were then incubated overnight with the primary antibodies diluted in 1% bovine serum albumin (Supplementary Table S1). After this period, the slides were immersed in 1 × PBS and incubated with the secondary biotinylated antibody (LSAB, DakoCytomation) in an oven for 30 min at 37 °C. The slides were again immersed in 1X PBS and incubated with streptavidin peroxidase (LSAB, DakoCytomation) for 30 min at 37 °C. The reactions were developed with 0.03% diaminobenzidine and 3% hydrogen peroxide as the chromogen solution. After this step, the slides were washed in distilled water and counterstained with Harris hematoxylin for 1 min. Finally, the sections were dehydrated in an increasing ethanol series and cleared in xylene.

### Quantitative analysis and photodocumentation

The portions removed from the samples were analyzed under an Axio Imager Z1 microscope (Zeiss, Oberkochen, Germany). Immunostaining was evaluated semiquantitatively by randomly selecting 30 fields at high magnification, including 10 in the meninges, 10 in the perivascular space, and 10 in the parenchyma in positive or negative cases. Each field was subdivided into 10 × 10 areas delimited by a grid, comprising an area of 0.0625 mm^2^. We highlight that for the meninges, three positive cases did not have this structure. For semiquantitative analysis, scores were attributed, taking into consideration the intensity of immunostaining in the tissue environment. The range adopted for each analysis is shown in Supplementary Table S2.

### Statistical analysis

The results were stored in electronic spreadsheets. Statistical analysis was performed with the GraphPad Prism 5.0 program. For univariate analysis, frequencies and measures of central tendency and dispersion were obtained. We highlight that for meninges only seven cases ZIKV-positive were included in the statistical analysis because presented structure and were compared to five controls. For space perivascular and parenchyma 10 cases ZIKV-positive were analized and compared to five controls. The hypotheses were tested using Student’s t (Supplementary Table S3) and Pearson’s correlation tests (Supplementary Tables S4–S6). A level of significance of 5% (*p* ≤ 0.05) was adopted for all tests.

## Electronic supplementary material

Supplementary Information
