# Supplementary material for: In situ immune response and mechanisms of cell damage in central nervous system of fatal cases microcephaly by Zika virus
Source: Sci Rep. 2018 Jan 8;8:1. doi: 10.1038/s41598-017-17765-5 (PMC5758755; doi:10.1038/s41598-017-17765-5)
Supplement: Supplementary file 1 — Supplementary Information [file 41598_2017_17765_MOESM1_ESM.pdf]

Supplementary Information

In situ immune response and mechanisms of cell damage in central nervous system of fatal cases microcephaly by Zika virus

Raimunda S.S. Azevedo[1], Jorge R. de Sousa[1], Marialva T. F. Araujo[3], Arnaldo J. Martins Filho[3], Bianca N. de Alcantara[2], Fernanda M. C. Araujo[4], Maria G. L. Queiroz[5], Ana C. R. Cruz[1,6], Beatriz H. Baldez Vasconcelos[7], Jannifer O. Chiang[1], Lívia C. Martins[1], Livia M. N. Casseb[1], Eliana V. da Silva[1], Valéria L. Carvalho[1], Barbara C. Baldez Vasconcelos[6], Sueli G. Rodrigues[1], Consuelo S. Oliveira[1,6], Juarez A. S. Quaresma[6,7], Pedro F. C. Vasconcelos[1,6*]

Supplementary Table and Legends

Supplementary Table S1. Antibodies used in the immunopathology study of fatal ZIKV microcephaly cases to characterize the immunopathogenesis congenital damage caused by Zika virus in the central nervous system.

Marker	Reference	Dilution
Caspase 3	Abcam (Ab 4051)	1:50
S100	Abcam (Ab 868)	1:200
CD68	Dako (PG M1 M0876)	1:50
CD163	Novocastra, NCL-L-CD163	1:50
CD57	Dako M 7271	1:50
CD8	Abcam (Ab 4055)	1:50
CD4	Abcam (Ab 51312)	1:50
FoxP3	Abcam (Ab 54501)	1:50
IFN-γ	R & D Systems MAB 285	1:50
IFN-α	Abcam (Ab 198914)	1:50
IFN-β	Abcam (Ab 140211)	1:50
IL-6	Abcam (Ab 154367)	1:100
IL-12A	Abcam (Ab 131039)	1:200
IL-1β	Abcam (Ab 9722)	1:50
TNF-α	Abcam (Ab 6671)	1:100
IL-4	Abcam (Ab 9622)	1:100
IL-10	Abcam (Ab ab18499)	1:200
IL-33	Abcam (Ab 18503)	1:50
IL-37	Abcam (Ab 116282)	1:50
TGB-β1	Abcam (Ab 66043)	1:50
IL-9	Abcam (Ab ab134434)	1:50
IL-17	Abcam (Ab 79056)	1:50
IL-23	Abcam (Ab 115759)	1:50
IL-22	Abcam (Ab 18499)	1:50
iNOS	Abcam (Ab 53769)	1:200
Arginase 1	Sigma HPA 003595	1:50

Supplementary Table S2. Semiquantitative immunohistochemistry scores for immunologic markers involved in the immune response against ZIKV.

Score	Intensity	%
0-1	Mild	0-25
1.1-2	Moderate	26-50
2.1-3	Intense	51-75
3.1-4	Very intense	76-100

Supplementary Table S3. Semiquantitative analysis of immunologic markers involved in ZIKV immunopathology. Student's t test; ns: not statistically significant; **p* < 0.05; ***p* < 0.005; ****p* < 0.0005.

	Meninges		Perivascular		Parenchyma	
Markers	Mean ± SD	p-value	Mean ± SD	p-Value	Mean ± SD	p-Value
Caspase 3	1.800 ± 0.680	0.0035**	1.509 ± 0.595	0.0007***	2.554 ± 0.961	0.0004***
Control	0.400 ± 0.547		0.200 ± 0.447		0.400 ± 0.548	
S100	2.229 ± 0.243	0.0004***	1.345 ± 0.539	0.0055**	2.545 ± 0.695	0.0060**
Control	1.200 ± 0.447		0.400 ± 0.547		1.400 ± 0.548	
CD68	0.443 ± 0.214	0.235	0.309 ± 0.446	0.6574	0.345 ± 0.425	0.5421
Control	0.200 ± 0.447		0.200 ± 0.447		0.200 ± 0.447	
CD163	1.929 ± 0.303	<0.0001***	2.073 ± 0.751	0.0002***	2.082 ± 0.805	0.0009***
Control	0.400 ± 0.547		0.200 ± 0.547		0.400 ± 0.548	
CD57	1.286 ± 0.380	0.0077**	1.709 ± 0.554	0.0006***	1.554 ± 0.528	0.0013**
Control	0.400 ± 0.547		0.400 ± 0.547		0.400 ± 0.548	
CD8	2.829 ± 0.325	<0.0001***	2.764 ± 0.544	<0.0001***	2.545 ± 0.650	<0.0001***
Control	0.600 ± 0.447		0.600 ± 0.547		0.600 ± 0.548	
CD4	2.057 ± 0.386	<0.0001***	2.045 ± 0.626	0.0007***	1.991 ± 0.577	<0.0001***
Control	0.400 ± 0.547		0.200 ± 0.447		0.400 ± 0.548	
FoxP3	2.157 ± 0.399	<0.0001***	2.500 ± 0.709	<0.0001***	2.564 ± 0.721	<0.0001***
Control	0.400 ± 0.547		0.400 ± 0.547		0.400± 0.548	
IFN-γ	0.971 ± 0.718	0.1673	1.591 ± 0.689	<0.0044**	2.036 ± 0.436	<0.0001***
Control	0.400 ± 0.547		0.400 ± 0.547		0.400 ± 0.548	
IFN-α	2.757 ± 0.391	<0.0001***	2.382 ±0.558	<0.0001***	2.627 ± 0.512	<0.0001***
Control	0.600 ± 0.547		0.400 ± 0547		0.400 ± 0.548	
IFN-β	2.157 ± 0.496	0.0002***	2.182 ± 0.513	<0.0001***	2.418 ± 0.538	<0.0001***
Control	0.400 ± 0.547		0.200 ± 0.447		0.400 ± 0.548	
IL-6	1.771 ±0.471	0.0049**	1.945 ± 0.410	<0.0001***	1.891 ± 0.559	0.0163*
Control	0.800 ±0.447		0.600 ± 0.547		1.000 ± 0.707	
IL-12A	2.543 ± 0.875	0.0014**	2.945 ± 0.789	<0.0001***	3.291 ± 0.539	<0.0001***
Control	0.600 ± 0.547		0.600 ± 0.547		0.400 ± 0.548	
IL-1β	2.085 ± 0.575	0.0012**	2.136 ± 0.951	0.0019**	2.154 ± 0.696	0.0006***
Control	0.600 ± 0.547		0.400 ± 0.547		0.600 ± 0.548	
TNF-α	1.786 ± 0.609	0.0061**	2.009 ± 0.777	0.0010**	2.482 ± 0.681	<0.0001***
Control	0.600 ± 0.547		0.400 ± 0.547		0.600 ± 0.548	
IL-4	2.714 ± 1.012	0.0018**	3.109 ± 0.709	0.0003***	3.436 ± 0.597	0.0003***
Control	0.600 ±0.547		0.600 ± 0.547		0.600 ± 0.548	
IL-10	3.543 ± 0.576	<0.0001***	3.182 ± 0.726	<0.0001***	3.527 ± 0.588	<0.0001***
Control	0.800 ± 0.447		0.800 ± 0.447		0.800 ± 0.447	
IL-33	3.971 ± 0.075	<0.0001***	3.809 ± 0.144	<0.0001***	3.873 ± 0.110	<0.0001***
Control	0.600 ± 0.547		0.600 ± 0.547		0.600 ± 0.548	
IL-37	2.729 ± 0.628	0.0002***	2.373 ± 0.665	<0.0001***	3.191 ± 0.427	<0.0001***
Control	0.600 ± 0.547		0.400 ± 0.547		0.600 ± 0.548	
TGF-β1	1.971 ± 0.904	0.0033**	1909 ± 0.947	<0.0020**	2.591 ± 0.709	<0.0001***
Control	0.400 ± 0.547		0.200 ± 0.447		0.200 ± 0.447	
IL-9	2.129 ± 0.379	<0.0001***	2.009 ± 0.230	<0.0001***	1.918 ± 0.419	<0.0001***
Control	0.400 ± 0.547		0.200 ± 0.447		0.600 ± 0.548	
IL-17	1.843 ± 0.588	0.0016**	2.145 ± 0.776	0.0005***	2.800 ± 0.467	<0.0001***
Control	0.400 ± 0.547		0.400 ± 0.547		0.800 ± 0.447	
IL-23	2.929 ± 1.029	0.0010**	2.736 ± 0.873	<0.0001**	3.436 ± 0.704	<0.0001***
Control	0.600± 0.547		0.400 ± 0.547		0.800 ± 0.447	
IL-22	1.771 ± 0.546	0.0016**	2.010 ± 0.746	<0.0021**	2.791 ± 0.597	<0.0001***
Control	0.400 ± 0.547		0.600 ± 0.547		0.800 ± 0.447	
iNOS	3.157 ± 0.588	<0.0001***	2.864 ± 0.827	0.0009***	3.545 ± 0.432	<0.0001***
Control	1.200 ± 0.447		1.200 ± 0.447		1.200 ± 0.447	
Arginase 1	3.043 ± 0.759	<0.0001***	2.682 ± 0.919	0.0002***	2.991 ± 0.729	<0.0001***
Control	0.400 ± 0.547		0.400 ± 0.547		0.400 ± 0.548	

Supplementary Table S4. Linear correlation between immunologic markers in the meninges in fatal ZIKV microcephaly cases. Pearson's correlation test; $p > 0.05$ = not statistically significant; $*p < 0.05$; $**p < 0.005$.

Correlation	r	p-value
CD4 x FoxP3	0,773	0,0411*
CD4 x CD68	0,7879	0,0353*
CD4 x IL-6	0,7681	0,0432*
CD4 xIL-12A	0,6711	0,098
CD4 x IL-17	0,7493	0,0525
CD4 x TNF-α	0,8173	0,024*
CD8 x IFN-γ	0,679	0,101
CD57 x IL-4	0,6831	0,0907
CD57 x IL-33	-0,9430	0,0014**
CD57 x IL-37	-0,8069	0,0283*
CD57 x TGF-β1	-0,6886	0,0871
CD68 x IL- 1β	0,7062	0,0760
CD68 x IL-6	0,8857	0,0080**
CD68 x IL-12A	0,8923	0,0069**
CD68 x IFN-γ	0,6340	0,1424
CD68 x Caspase 3	0,6608	0,1061
CD163 x iNOS	-0,7562	0,0492*
S100 x IL-4	-0,8014	0,0302*
S100 x IL-22	-0,7203	0,067
S100 x Caspase 3	-0,6348	0,1255
IL- 1β x IL-12 A	0,6331	0,1269
IL- 1β x IL-22	0,6173	0,1331
IL- 1β x IL-23	0,7744	0,0409*
IL- 1β x TNF-α	0,7119	0,0727
IL- 1β x INF-Y	0,7400	0,0568
IL- 1β x Caspase 3	0,6933	0,0841
IL-4 x IL -37	0,904	0,0052**
IL-4 x TGF-β1	0,8627	0,0124*
IL-4 x Arginase 1	0,6504	0,113
IL-4 x Caspase 3	0,9022	0,0052**
IL-6 xIL-12A	0,7625	0,0462*
IL-6 x TNF-α	0,7058	0,0763
IL-10 x TGF-β1	0,7634	0,0458*
IL-10 x Caspase 3	0,5263	0,2249
IL-12A x IL-22	0,7028	0,0781
IL-12A x TNF-α	0,8730	0,0103*
IL-12A x IFN-Y	0,7553	0,0496*
IL-12A xIFN–α	0,7027	0,0078**
IL-12A x Caspase 3	0,7972	0,0317*
IL-17 x TNF- α	0,7642	0,0454*
IL-17 x Caspase 3	0,7948	0,0326*
IL-22 x IFN-Y	0,7827	0,0374*
IL-22 x Caspase 3	0,8507	0,0035**
IL-33 x IL-37	0,7282	0,0629*
IL-37 x TGF-β1	0,8794	0,0091**
IL-37 x Caspase 3	0,8862	0,0079**
TNF-α x Caspase 3	0,8557	0,0013**
TGF-β1 x Caspase 3	0,809	0,0273*
IFN-γ x IFN-α	0,7607	0,0470*

Supplementary Table S5. Linear correlation between immunologic markers in the perivascular space in fatal ZIKV microcephaly cases. Pearson's correlation test; $p > 0.05$ = not statistically significant; *$p < 0.05$; **$p < 0.005$; ***$p < 0.0005$.

Correlation	r	p-value
CD8 x TNF-α	0,6218	0,0410*
CD57 x TNF-α	0,7540	0,0073**
CD57 x Caspase 3	0,6432	0,0327*
FoxP3 x Caspase 3	0,5871	0,0575
CD68 x IL- 37	-0,5950	0,0530
CD68 x iNOS	0,5809	0,0608
CD163 x IL-10	0.8806	0,0003***
CD163 x Arginase 1	0,6160	0,0435*
S100 x IL-9	-0,5945	0,0537
IL-1β x IL-6	0,6354	0,0356*
IL-1β x IL-17	0,5976	0,0521
IL-1β x IL-23	0,6511	0,0300*
IL-4 x IL-10	0,8112	0,0024**
IL-4 x TGF-β1	0,6242	0,0400*
IL-4 x Arginase 1	0,8570	0,0007***
IL-4 x IL-37	0,7351	0,0099**
IL-6 x IL-17	0,6044	0,0488*
IL-6 x IL-23	0,6166	0,0433*
IL-6 x iNOS	0,7880	0,0040**
IL-9 x IL-22	0,6228	0,0406*
IL-9 x TNF-α	0,6479	0,0311*
IL-9 x IFN-γ	0,7450	0,0025**
IL-9 x IFN-α	0,6244	0,0399*
IL-9 x IFN-β	0,9414	<0,0001***
IL-10 x TGF-β1	0,6530	0,0293*
IL-10 x Arginase 1	0,8213	0,0019**
IL-12A x IFN-β	0,6908	0,0185*
IL-12A x IL-22	0,7702	0,0055**
IL-12A x IFN-γ	0,6260	0,0353*
IL-17 x TNF-α	0,6882	0,0192*
IL-17 x IFN-α	0,5882	0,0569
IL-17 x iNOS	0,7532	0,0074**
IL-22 x IFN-β	0,6268	0,0390*
IL-22 x iNOS	0,8071	0,0027**
IL-23 x TNF-α	0,7650	0,0068**
IL-23 x IFN-α	0,7277	0,0111*
IL-23 x iNOS	0,6636	0,0259*
TNF-α x IFN-α	0,8618	0,0006***
TNF-α x IFN-β	0,6590	0,0274*
TNF-α x iNOS	0,6284	0,0383*
TNF-α x Caspase 3	0,6332	0,0364*
TGF-β1 x Arginase 1	0,7626	0,0063**
TGF-β1 x Caspase 3	0,6389	0,0343*
IFN-α x IFN-β	0,7314	0,0105*
IFN-α x Caspase 3	0,7346	0,0100*
IFN-γ x IFN-α	0,6312	0,0372*
IFN-γ x IFN-β	0,6665	0,0251*
IFN-γ x Caspase 3	0,6317	0,0376*
IL-33 x CD163	0,6102	0,046*
IL-33 x IL-10	0,6777	0,0219*
IL-33 x TGF β1	0,7515	0,0076**

Supplementary Table S6. Linear correlation between immunologic markers in the neural parenchyma in fatal ZIKV microcephaly cases. Pearson's correlation test; $p > 0.05$ = not statistically significant; *$p < 0.05$; **$p < 0.005$; ***$p < 0.0005$.

Correlation	r	p-value
CD4 x FoxP3	0,777	0,004**
CD4 x IL-1β	0,638	0,034*
CD4 x IL-4	0,619	0,042*
CD4 x IL-10	0,716	0,013*
CD4 x IL-12A	0,632	0,036*
CD4 x IL-17	0,690	0,018*
CD4 x IL-23	0,636	0,035*
CD4 x TNF-α	0,661	0,035*
CD4 x IFN-α	0,657	0,026*
CD57 x IFN-α	0,700	0,016*
CD57 x IFN-β	0,573	0,065
FoxP3 x IL-10	0,641	0,033*
FoxP3 x IL-12A	0,631	0,044*
CD163 x IL-33	0,658	0,027*
CD163 x IFN-γ	-0,601	0,050*
IL-1β x Il-12A	0,779	0,004**
IL-1β x IL-17	0,670	0,023*
IL-1β x TNF-α	0,618	0,042*
IL-1β x Caspase 3	0,599	0,051
IL-4 x IL-10	0,805	0,002*
IL-4 x IL-33	0,744	0,008*
IL-4 x IL-37	0,823	0,001**
IL-4 x Arginase 1	0,575	0,063
IL-4 x Caspase 3	0,688	0,019*
IL-10 x IL-33	0,628	0,038*
IL-10 x IL-37	0,688	0,019*
IL-10 x Arginase 1	0,751	0,007**
IL-10 x Caspase 3	0,646	0,031*
IL-12A x IL-17	0,603	0,049*
IL-12A x TNF-α	0,846	0,001**
IL-12A x IFN-α	0,627	0,038*
IL-17 x IL-23	0,653	0,029*
IL-17 x TNF-α	0,701	0,016*
IL-17 x IFN-α	0,715	0,013*
IL-17 x IFN-β	0,589	0,056
IL-17 x Caspase 3	0,729	0,010*
IL-23 x IFN-α	0,677	0,024*
IL-33 x IL-37	0,611	0,030*
IL-33 x Caspase 3	0,609	0,046*
IL-37 x Arginase 1	0,644	0,032*
IL-37 x Caspase 3	0,619	0,042*
TNF-α x IFN-α	0,767	0,005**
TNF-α x IFN-β	0,617	0,042*
TNF-α x Caspase 3	0,666	0,025*
IFN-α x IFN-β	0,890	0,0002***
TGF-β1 x Caspase 3	0,684	0,020*

Supplementary Table S7. **Distribution of 10 fatal Zika virus (ZIKV) microcephaly cases and five control samples according to category, sex, and results of specific ZIKV immunohistochemistry (IHC) and real-time PCR (RT-qPCR). M**: male; **F**: female; **IHC**: immunohistochemical assay; **RT-qPCR**: quantitative real-time reverse transcription polymerase chain reaction; **Pos**: positive; **NR**: not realized; **Neg**: negative.

Case	Category	Lifetime	Sex	Case information	IHC (ZIKV)	RT-qPCR (ZIKV)
				**Microcephaly**		
**1**	Newborn	6 hours	F	Microcephaly with ventriculomegaly, arthrogryposis, pulmonary hypoplasia, atrial septal defect	Pos	Pos
**2**	Newborn	2 hours	M	Newborn with multiple malformations: microcephaly, large anterior fontanelle, nasopalatine palate, low-set ears, ginodactilia, clubfoot	Pos	Neg
**3**	Newborn	27 days	M	Microcephaly detected by ultrasonograph. During necropsy procedures observed anophthalmia, holoprosencephaly, agenesis of cerebellar vertix, hypoplasia and poor pulmonary formation.	Pos	Pos
**4**	Newborn	1 day	F	Microcephaly and bilateral ventriculomegaly detected on 35 weeks of gestacional age. Cesarean delivery	Pos	NR
**5**	Newborn	2 days	M	Microcephaly	Pos	Neg
**6**	Newborn	1 day	M	Microcephaly Unknown	Pos	Pos
**7**	Newborn	14 hours	M	Newborn with microcephaly, swing feet, upper limbs with arthrogryposis and hands back. Macroscopic pulmonary hypoplasia, little brain tissue, cerebellar hypoplasia, testicles in the abdominal cavity, thoracolumbar scoliosis.	Pos	Pos
**8**	Newborn	1 day	M	Ultrasonography at 22 weeks detected microcephaly, congenital clubfoot, agenesis of fingers and collapse of the stomach. At birth microcephaly, deformity of the hands and feet, sketch of the scrotum.	Pos	Pos
**9**	Stillbirth	NA	M	Macroscopic aspects: brain malformed, dilated lateral ventricles and the absence of the cerebellum; absence of nasal bone, clubfoot; bilateral cubital fold; absence of scrotum; hypoplastic penis; bilateral cryptorchidism.	Pos	Pos
**10**	Stillbirth	NA	F	Microcephaly detected at birth.	Pos	NR
				**Controls**		
**11**	Newborn	10 days	F	No microcefaly. Death ten days after birth with cyanosis and ecchymosis after feeding.	Neg	Neg
**12**	Stillbirth	NA	M	Microcephaly case without evidence of ZIKV infection.	Neg	Neg
**13**	Stillbirth	NA	F	Microcephaly case without evidence of ZIKV infection.	Neg	NR
**14**	Stillbirth	NA	F	Microcephaly case without evidence of ZIKV infection. Birth with 31 weeks of pregnancy.	Neg	NR
**15**	Newborn	19 hours	F	Microcephaly case. Mother with granulomatous disease, hypertensive disease of pregnancy and hyperthyroidism, but no evidence of infectious disease during pregnancy.	Neg	Neg
